# Population‐level manipulations of field vole densities induce subsequent changes in plant quality but no impacts on vole demography

**DOI:** 10.1002/ece3.4204

**Published:** 2018-07-13

**Authors:** Lise Ruffino, Susan E. Hartley, Jane L. DeGabriel, Xavier Lambin

**Affiliations:** ^1^ School of Biological Sciences University of Aberdeen Aberdeen UK; ^2^ Department of Biology University of York York UK; ^3^ NSW Office of Environment and Heritage Sydney NSW Australia

**Keywords:** density‐dependence, induced defense, *Microtus agrestis*, population cycles, silicon

## Abstract

Grazing‐induced changes in plant quality have been suggested to drive the negative delayed density dependence exhibited by many herbivore species, but little field evidence exists to support this hypothesis. We tested a key premise of the hypothesis that reciprocal feedback between vole grazing pressure and the induction of anti‐herbivore silicon defenses in grasses drives observed population cycles in a large‐scale field experiment in northern England. We repeatedly reduced population densities of field voles (*Microtus agrestis*) on replicated 1‐ha grassland plots at Kielder Forest, northern England, over a period of 1 year. Subsequently, we tested for the impact of past density on vole life history traits in spring, and whether these effects were driven by induced silicon defenses in the voles’ major over‐winter food, the grass *Deschampsia caespitosa*. After several months of density manipulation, leaf silicon concentrations diverged and averaged 22% lower on sites where vole density had been reduced, but this difference did not persist beyond the period of the density manipulations. There were no significant effects of our density manipulations on vole body mass, spring population growth rate, or mean date for the onset of spring reproduction the following year. These findings show that grazing by field voles does induce increased silicon defenses in grasses at a landscape scale. However, at the vole densities encountered, levels of plant damage appear to be below those needed to induce changes in silicon levels large and persistent enough to affect vole performance, confirming the threshold effects we have previously observed in laboratory‐based studies. Our findings do not support the plant quality hypothesis for observed vole population cycles in northern England, at least over the range of vole densities that now prevail here.

## 1 INTRODUCTION

Delayed density‐dependence drives multiannual cyclic fluctuations in abundance of many herbivore populations, such that current population densities are partly regulated by past ones. This phenomenon has been well‐documented, but there is still considerable debate about the underlying mechanisms. Whereas hypotheses based on specialist predation are well supported, at least for some systems (e.g., Hanski, Hannson, & Henttonen, 1991; Gilg, Hanski, & Sittler, 2003; but see Lambin, Krebs, Moss, & Yoccoz, 2002; Lambin, 2017), little empirical support exists for negative feedback between herbivore density and food availability (Turchin & Batzli, 2001). While the negative impacts of herbivore density on the quantity of food may not be evident, except in arctic and subarctic systems, where plant regrowth after herbivory is slow (Krebs, Cowcill, Boonstra, & Kenney, 2010; Turchin, Oksanen, Ekerholm, Oksanen, & Henttonen, 2000), it is now well established that herbivory‐induced reductions in plant quality (termed induced defences; Karban & Myers, 1989) do have the potential to underlie the delayed density dependence of cyclic herbivore populations (Reynolds et al., 2012; Turchin, 2003; Underwood & Rausher, 2002). However, thus, far there is relatively little population‐scale field evidence to support this hypothesis, as previous studies have generally been either theoretical (e.g., Kent, Jensen, & Doncaster, 2005; Turchin, 2003; Underwood, 1999) or based on laboratory experiments or field enclosures (Huitu, Koivula, Korpimäki, Klemola, & Norrdahl, 2003; Huitu et al., 2014; Klemola, Koivula, Korpimaki, & Norrdahl, 2000; Reynolds et al., 2012).

The population dynamics of most grass‐feeding vole species, in particular those of the genus *Microtus*, are driven by delayed density‐dependent processes (e.g., Bjørnstad, Falck, & Stenseth, 1995). Demographically, this can be mediated by variation in the timing of onset of their spring reproduction, which is delayed by high population densities in the previous year (Ergon, Ergon, Begon, Telfer, & Lambin, 2011; Pinot et al., 2016). Theoretical studies have suggested that such density‐dependent impacts on breeding season length alone have the potential to generate population cycles in seasonal environments (Smith, White, Lambin, Sherratt, & Begon, 2006), while in the field, voles transplanted at the start of winter between grassland areas differing in the phase of their cycle have been shown to take on the characteristics of vole populations in their new environment (Ergon, Lambin, & Stenseth, 2001). This demonstrates that the mechanisms driving vole demography must arise from interactions within their immediate environment (also see Klemola, Korpimäki, & Koivula, 2002). Grazing‐induced changes in grass quality have been suggested as a possible underlying mechanism to explain this observed impact of the local environment on voles (Massey & Hartley, 2006; Massey, Smith, Lambin, & Hartley, 2008; Reynolds et al., 2012; Wieczorek, Zub, Szafrańska, Książek, & Konarzewski, 2015).

Grasses (*Poaceae*), the main food source for *Microtus* voles (Stenseth, Hansson, & Myllymäki, 1977), accumulate silicon in their leaves to deter herbivore feeding (Massey, Ennos, & Hartley, 2006; Massey, Massey, Ennos, & Hartley, 2009; Reynolds, Keeping, & Meyer, 2009). Silicon is taken up from the soil and actively transported, primarily to the leaves, where it is deposited as abrasive phytoliths (Jernvall & Fortelius, 2002; Massey et al., 2006). Silicon levels have been correlated with intensity of mammal grazing in ecosystems as diverse as the Serengeti in East Africa, temperate grasslands in northern England and arctic riparian meadows in Norway (Massey et al., 2008; McNaughton, Tarrants, McNaughton, & Davis, 1985; Soininen, Bråthen, Herranz Jusdado, Reidinger, & Hartley, 2013; Wieczorek, Zub, et al., 2015).

In voles, evidence compatible with a possible reciprocal negative feedback between grazing and silicon induction has been observed under laboratory conditions; high levels of vole grazing increased silicon levels by up to 400% (Garbuzov, Reidinger, & Hartley, 2011; Massey, Ennos, & Hartley, 2007b), in turn significantly reducing vole growth rates, possibly because silicon impeded voles’ ability to extract nitrogen from food (Massey & Hartley, 2006). More recently, the abrasive properties of silicon phytoliths have also been shown to increase tooth wear in voles (Calandra, Zub, Szafrańska, Zalewski, & Merceron, 2016), as well as damage their small intestine, reducing body mass and metabolic rate (Wieczorek, Szafrańska, Labecka, Lázaro, & Konarzewski, 2015). Population models incorporating the observed silicon induction response, and the assumption that the empirical relationship between past vole density and timing of onset of vole spring reproduction (Ergon et al., 2011) is mediated by leaf silicon concentrations, consistently predicted cyclic changes in vole population densities (Reynolds et al., 2012). However, it remains unclear whether the amplitude of herbivore‐induced changes in silicon concentrations observed under controlled conditions are replicated in the field (but see Hartley & DeGabriel, 2016), nor is it known whether such changes are of sufficient magnitude and duration to affect vole demography, specifically the onset of reproduction in spring, in wild *Microtus* vole populations. Thus, manipulative field experiments that test for functional links between grazing pressure, silicon induction and vole growth and reproduction under ecologically relevant conditions are needed to assess potential impacts of silicon defenses on vole populations.

We carried out a large‐scale field experiment to test how pulsed reduction of densities of natural vole populations affected leaf silicon concentrations in their major winter food plant *Deschampsia caespitosa* and whether this influenced vole demography, in particular the timing of onset of spring reproduction. Previous attempts to disentangle the role of plant quality in the population cycles of voles have commonly used enclosures to either exclude or confine vole populations, often over relatively small time‐scales (Huitu, Laaksonen, Norrdahl, & Korpimäki, 2005; Huitu et al., 2014; Klemola, Koivula, et al., 2000; Soininen et al., 2013). Some of these enclosure‐based approaches have revealed the effects of food availability or quality on key demographic parameters and/or demonstrated effects on silicon levels in plants, but the most ecologically meaningful test of whether silicon induction drives population cycles is to manipulate vole densities at the landscape scale and observe the effects on plant quality and vole demography in subsequent years. This was attempted in a recent study on root voles feeding on sedges (Wieczorek, Zub, et al., 2015), using two 1‐ha enclosures to confine populations and monitoring effects on silicon levels and vole performance within the fences. Our novel approach here is to attempt a replicated experimental test of the link between silicon induction and vole populations designed to break down any confounding effects between natural variation in density and other variables affecting populations. We manipulated the trajectories of natural populations in the field by creating areas of high and low vole densities and observing the impact on silicon induction and vole performance over subsequent years and at the landscape scale.

We predicted that (a) leaf silicon concentrations in *D*. *caespitosa* would be greatest on sites with high vole population densities. As vigorously growing, early‐season foliage has a greater capacity to respond to damage than does late‐season foliage (Karban & Baldwin, 1997; Nykanen & Koricheva 2004), we predicted that (b) silicon induction would be greatest between early spring and summer. Following the findings of Reynolds et al. (2012), we predicted that silicon concentrations would diverge between high and low population density sites after a delay of several months and that leaf silicon concentrations would remain elevated for several months after the cessation of the density manipulations.

Ergon et al. (2011) demonstrated that the mean date of onset of vole reproduction in spring was delayed by about 24 days for every additional 100 voles/ha in the previous spring. We therefore predicted that (c) vole populations over‐wintering on sites with previously higher densities would gain mass more slowly, start to breed later in spring and reach higher densities than voles over‐wintering on sites with previously low densities and that this effect would reflect differences in leaf silicon concentrations. From our previous work (Massey & Hartley, 2006), leaf silicon levels over ~1.5% dry weight are required to affect vole growth rates. Hence, we expected these impacts on vole mass and breeding would occur once silicon levels on the control sites exceed these levels.

## 2 MATERIALS AND METHODS

### 2.1 Study area

The experiment was carried out in Kielder Forest, a 600 km^2^ upland forest‐grassland ecosystem in northern England (55°13′N, 2°33′W). Field vole populations at Kielder show cyclic dynamics with a 3–4 year periodicity and population densities of 20 to 765 voles per hectare (Ergon et al., 2011; Lambin, Petty, & MacKinnon, 2000). Populations situated close together fluctuate in synchrony, but cycles can be asynchronous at a wider spatial scale (Lambin, Elston, Petty, & Mackinnon, 1998).

### 2.2 Experimental design

We established six permanent trapping grids (Ugglan Special Mousetraps, Grahnab, Marieholm, Sweden) on three pairs of grass‐dominated forest clear‐cut sites in Kielder Forest (three experimental and three control grids). Given that our aim was to test the effects of silicon induction in *D. caespitosa*, we selected sites such that this was the dominant food plant available to voles and presumably comprised the main part of their diet. In late spring 2010, we abandoned the original control site of pair 3 because, consistent with earlier farming activities, the vegetation showed signs of eutrophication (i.e., growth of nitrophilous plants such as *Dactylis glomerata*,* Urtica dioica,* and *Bromus* sp.), and thus, evidence of feeding damage on the relatively unpalatable foliage of *D*. *caespitosa* was very low, despite high vole population densities. We established a replacement site about 1 km away and commenced collecting plant samples in June 2010 and trapping to estimate vole density in August 2010. Because vole populations situated close together in Kielder Forest fluctuate in synchrony (Lambin et al., 1998), we supplemented the vole density estimates for this new control site with a density estimate taken at the end of September 2010 on a *D*. *caespitosa* dominated site <500 m away. Supporting Information Table S1 lists the dates when plant samples were taken, voles were removed and population densities recorded.

During the *induction* phase of our experiment, we regularly reduced field vole densities on one site within each pair (the “removal site”), but did not manipulate vole densities on the “control sites” (Supporting Information Table S1). We started the *induction* phase of the experiment during a field vole population peak in October 2009 and finished it in November 2010, by which time population densities in the areas had declined. In November 2010, we carried out a field transplant experiment, where we trapped and removed all adult voles from all six sites. We then added “naïve,” non‐reproducing voles to each site, in order to achieve similar densities across all sites, such that no vole was replaced on its site of origin. During the *response* phase, from November 2010 until June 2011, we trapped voles monthly to test whether past differences in vole densities between control and removal sites, and their potential impact on plant quality, affected their demography. Our experimental design insured that any observed differences in population dynamics between sites could be ascribed to their current environment and were not biased by differences in vole quality within sites.

### 2.3 Trapping

During the *induction phase*, we placed 100 traps at 5‐m intervals over an area of 0.3 ha on the control sites and estimated field vole population densities using capture–mark–recapture techniques. We checked the traps morning and evening for five consecutive trapping sessions (i.e., over 2.5 days) at irregular intervals between March and September 2010 (Supporting Information Table S1). We individually marked trapped voles with ear tags before releasing them at the point of capture. The trapping grids on the removal sites were larger (0.93 ± 0.09 ha; ~200 traps at 7 m intervals, with traps in adjacent lines staggered), and we released all voles caught at forest clear‐cuts away from the experimental grids. We trapped the removal sites, usually for four consecutive days in most months between October 2009 and October 2010 (Supporting Information Table S1) to insure that all voles were caught and removed. If new voles were still being caught after 4 days, additional trapping days were included at those sites until we were confident that we had removed all voles. To supplement our density estimates from trapping, we regularly estimated vole population densities at each of the six sites using a calibrated Vole Sign Index (VSI) based on the presence of grass clippings (Lambin et al., 2000).

In November 2010, we enlarged the trapping grids on the control sites to approximate the removal sites (0.77 ± 0.11 ha) and removed all voles from all sites. We weighed all voles caught (±0.5 g) using a digital balance and determined their reproductive status. Females were classified as reproductive when they were lactating (enlarged nipples), and/or their pubic symphysis or vaginal opening indicated that they had recently given birth and we only included apparently virgin females in the experiment. We marked all voles used with subcutaneous Passive Integrated Transponder (PIT) tags (Trovan Ltd., UK). To supplement the low number of virgin females caught on the removal and control sites during the transplant, we also transplanted virgin females caught on nonexperimental sites. Each individual was randomly allocated to one of the sites (removal or control, but not the site of their capture), insuring that the sex ratio was kept similar between sites (27.8 ± 0.48 male and 15.7 ± 0.76 female voles per site, Supporting Information Table S1).

From January 2011 onwards, we trapped the sites at monthly intervals to record the mass and reproductive status of the marked voles. Voles immigrating onto the trapping grids from the surrounding areas were also PIT‐tagged and released at the site of capture.

### 2.4 Plant quality and vegetation measurements

In November 2009, the vegetation on the six experimental sites was dominated by the grasses *D. caespitosa* (35.6 ± 6.0%), *Holcus lanatus* (23.5 ± 6.7%), *Agrostis capillaris* (8.4% ± 3.4) and *Festuca ovina* (3.3 ± 2.1%). The rush *Juncus effusus* (13.3 ± 5.4%) and several forb species (e.g., *Epilobium spp., Digitalis purpurea*,* Ranunculus acris* and *Rumex acetosa*, all <2% cover) were also present. Because *D*. *caespitosa* remains green throughout the year (Davy, 1980), whereas leaves of other species quickly die back after prolonged periods of frost and snow cover, *D*. *caespitosa* constitutes the most important over‐winter food source for herbivores in such mesotrophic grassland communities (Rodwell, 1998; also see Stenseth et al., 1977; Klemola, Norrdahl, & Korpimaki, 2000). This is supported by our VSI surveys in March 2010, which showed that typically >80% of all grass clippings produced by field voles were *D*. *caespitosa*.

At monthly or bimonthly intervals from October 2009 until June 2011 (Supporting Information Table S1), we collected 5–8 tillers from each of 10 *D*. *caespitosa* tussocks randomly selected from across each of the six 0.3–0.9 ha trapping sites by walking 30 paces between tussocks. New tussocks were selected on each sampling visit. The pooled tillers from each sampling location were stored in sealed plastic bags at −18°C. We washed the leaves thoroughly under running tap water, dried them in a fan‐assisted oven at 70°C for 3 days, and ground them using a Pulverisette 23 ball mill (Fritsch GmbH, Germany). We analyzed silicon concentrations with a portable X‐ray fluorescence spectrometer using the method described by Reidinger, Ramsey & Hartley (2012). We also analyzed nitrogen and carbon concentrations using flash combustion followed by gas chromatographic separation (Elemental Combustion System; Costech Instruments, Milan, Italy). A freezer failure affected the samples collected between December 2009 and April 2010, so they were not analyzed (Supporting Information Table S1).

## 3 DATA ANALYSIS

### 3.1 Estimation of population density

We estimated vole population size at each site using closed capture models in program MARK (White & Burnham, 1999), fixing the recapture probability to zero for the removal data during the *induction phase*, and selected the best models based on Akaike's Information Criterion, corrected for small sample size (AICc; Burnham & Anderson, 1998). For each site and trapping session, vole population density was estimated by dividing estimates of population size by effective trapping areas, accounting for nonhabitats such as roads and rivers and a buffer strip of one trap spacing width where habitat extended beyond the grid. For each site, we estimated average vole densities during the spring months of the *induction phase* (e.g., 15 March–31 May 2010) as the density obtained for the midpoint between sampling intervals (i.e., 22 April 2010) using linear interpolation. On three occasions during the *induction phase,* we were not able to estimate population sizes on the removal sites with MARK, due to a lack of vole depletion over the course of the trapping session (removal site Pair A: June 2010; removal site Pair B and C: October 2010). For these sites and time points, we roughly estimated population sizes by dividing the sum of all animals caught by the capture probability for that site during the preceding trapping session.

### 3.2 Effects of grazing on silicon induction

First, we tested how vole density manipulations affected leaf silicon concentrations in *D*. *caespitosa* during the *induction* phase. We performed a linear mixed effects model (LMM) on the response variable “silicon concentration,” where a “pair/plot/month” random structure was specified to account for the nested design of our experiment. We predicted that the strongest treatment effects on silicon concentration would occur from early summer onwards and accounted for this in our models by including “period” as a second fixed effect (winter/spring: 24/09/09–04/04/10; summer/autumn: 11/06/10–16/11/10) and the interaction term “treatment*period.” Second, we tested how vole density manipulations during spring of the *induction* phase affected silicon concentrations during the subsequent vole *response* phase. Because voles are expected to be most nutritionally limited as they are preparing to enter spring reproduction (Ergon, Speakman, Scantlebury, Cavanagh, & Lambin, 2004; Speakman et al., 2003), we focused on silicon concentrations in *D*. *caespitosa* in February and March 2011. We performed LMM with “treatment” and “month” included as fixed effect, with a nested “pair/plot” random structure. For both analyses, we compared model performance between those including “treatment” as a categorical variable (control vs. removal) and those including vole densities during spring of the *induction* phase as a continuous variable (estimated as densities on 22 April 2010; see above), using AICc. In both cases, models including “treatment” performed better (see Supporting Information Table S2).

**Figure 1:**
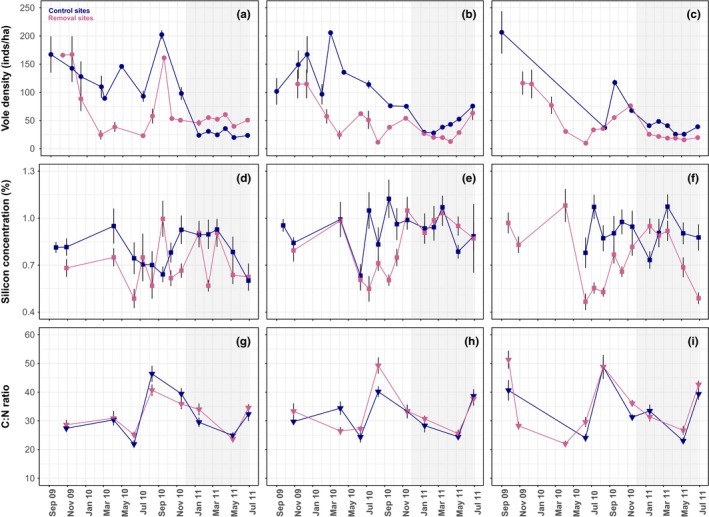
Changes in field vole population densities (a–c), leaf silicon concentrations (d–f), and leaf carbon‐nitrogen ratios (g–i) over the induction and response phase of the experiment. Black arrows on the *X* axis indicate the start of the response phase (highlighted in gray). Average values (±*SE*) for removal and control sites are represented by pink and dark blue symbols, respectively. Vole density estimates shown (a–c) are from both VSI survey (September 2009–February 2010) and trapping data (March 2010 onwards)

Third, we tested whether vole density manipulations affected leaf carbon–nitrogen ratios (C:N) using a LMM with “treatment” as fixed effect and a nested “pair/plot” random structure. We added “month” as a non‐nested random effect as visualization of real C:N data revealed apparent similar monthly variation across pairs and plots (see Figure 1g–i). As we had no a priori expectations of how nitrogen might respond to grazing, and how this might be influenced by season, analyses were performed separately for the *induction* (24/09/09–16/11/10) and *response* phase (14/01/11–26/06/11). Response variables were log‐transformed prior analyses in cases where the assumption of normality of residuals was not met.

### 3.3 Effect of past grazing on future vole performance

We restricted our analyses to vole performance between March and May 2011, spanning the earliest and latest date when overwintered females were postpartum for the first time. We tested whether female body mass, timing of onset of breeding, and population growth rates varied between treatments. At each capture, we scored each female as either pre‐ or postpartum. For those females caught repeatedly, we also considered changes in body mass as an indicator of reproduction. Because of the small sample size at some sites, we also included females that immigrated onto the sites after the transplant and were first caught in winter or early spring 2011 under the assumption their performance was affected by local grass quality. We excluded all females from our analyses that had seemingly given birth in the previous year (semi‐open pubic symphysis), or whose previous breeding history we were unable to confidently assess so that we could attribute any observed effects on female reproductive success to their foraging environment and avoid any confounding effects of previous maternal investment on future breeding. Sample sizes are given in Supporting Information Table S1.

In March 2011, 0% (*n* = 29) and 13% (*n* = 23) of females caught in removal and control sites, respectively, were scored as reproductively mature. We estimated whether the likelihood of females being postpartum mid‐spring of the response phase (April 2011) differed between control and removal sites. We used a generalized linear mixed effects model with “treatment” as fixed effect and a “pair/plot” nested random structure. We did not model the probability of being reproductively mature between March and June 2011 due to some group variables (treatment*date) having only zeros or ones. The effect of past vole density manipulations on female body mass was modeled using a LMM including “treatment*month” as fixed effect and a “pair/plot/voleID” random structure. We compared the performance between models including “treatment” as a categorical variable and “average spring vole densities” as a continuous variable, using AICc. Finally, we calculated spring population growth rates as ʎ = log (*D*_June11_ + 1) − log(*D*_March11_ + 1) and tested whether ʎ differed between treatments using a paired sample *t*‐test.

### 3.4 Vole survival

In order to test whether vole density manipulations influenced future vole survival, we estimated apparent survival (which reflects the probability of surviving and remaining on the trapping grid) and recapture probabilities during the *response phase* only (November 2010–May 2011) using standard open Cormack–Jolly–Seber models (Lebreton, Burnham, Clobert, & Anderson, 1992) implemented in MARK. Survival probabilities refer here to the probability of survival for a 28‐day period. We started the analyses by selecting a global, fully time‐dependent model where the probabilities of survival *ɸ* and recapture *p* depend on sex, pair, and treatment. We carried out initial goodness‐of‐fit tests for this model using the parametric bootstrap procedure in MARK and calculated the quasi‐likelihood parameter (“c‐hat” = 1.15). Our model selection process followed standard procedures (Lebreton et al., 1992) to increase the power of detecting variation in survival, we first modeled the variation in *p* before constraining variation in *ɸ*. We used conditional AICc to compare the goodness‐of‐fit among models. Models were ranked in relation to each other using ΔAICc values. AICc weights were calculated to assess the relative likelihood of each model considered (Cooch & White, 2014). In our candidate model set, *p* varied with sex, pair, and treatment and the time structure was fully time‐dependent, constant, or dependent on the presence of snow cover during the trapping session. The model where *p* depended on pair and snow cover was best supported by the data. We then modeled the variation in survival, where *ɸ* varied with sex, pair, and treatment. As we had no a priori predictions on how the treatment may impact on survival rates over time, the time structure was either fully time dependent, constant, or dependent on the reproductive season (nonreproductive season: November–March; reproductive season: April–May).

### 3.5 Protection of human subjects and animals in research

The work complied will all legal requirements in England. Specifically, no invasive procedure was performed and as such, the work did not fall under the remit of the Animals (Scientific Procedures) Act 1986. Ugglan Live Traps either had a shrew escape hole or were modified so as to avoid shrew capture.

## 4 RESULTS

### 4.1 Vole population densities during the *induction* phase

Consistent with 3–4 year cycles in vole abundance, mean population densities on the control sites decreased over the summer/autumn of 2010, from 124.0 (±23.7 *SE*) voles/ha in April, to 80.3 (±15.8 *SE*) voles/ha before the vole transplant in November. Between October 2009 and February 2010 in the *induction* phase, we removed a cumulative total of 377 voles/ha from the removal site of Pair A, 197 voles/ha from the removal site of Pair B, and 413 voles/ha from the removal site of Pair C (in four, three, and four trapping sessions, respectively; Supporting Information Table S1). As expected, vole densities consistently recovered, at least partially, over the course of the month that separated pulsed removals through immigration from the surroundings. This recovery meant densities at time of trapping on the removal sites varied greatly over time, from 9.90 (±0.01 *SE*) voles/ha in June to 161.21 (±2.82 *SE*) voles/ha in November 2010 (Figure 1a–c). Between 15 March and 31 May 2010, densities on the removal sites were 68, 123, and 132 voles/ha lower than those on the corresponding control sites (or 70, 88, and 98% for pairs A, B, and C, respectively; Figure 1a–c).

### 4.2 Plant chemistry

Mean leaf silicon concentrations of *D*. *caespitosa* ranged between 0.47 ± 0.05% (removal site of pair 3, June 2010) and 1.12 ± 0.12% (control site of pair 2, September 2010; Figure 1d–f). During the second period of the *induction* phase (June–November 2010), plants on the control sites had higher leaf silicon concentrations than those on the removal sites. While silicon concentrations between October 2009 and April 2010 were only 2.5% lower on the removal than on the control sites, this difference increased to 22.1% over the following summer and autumn (treatment*period: *β* = −0.04, *SE* = 0.02, *t* = −2.00; Figure 1d–f). With every increase in early‐season grazing pressure by 100 voles/ha, silicon concentrations between June and November 2010 increased by 0.15 ± 0.03% (*t*_spring_densities_ = 5.47). The vole density manipulations had no enduring significant effect on leaf silicon concentrations during early spring (February and March) of the vole *response* phase (treatment: *β* = −0.09, *SE* = 0.05, *t* = −1.65; Figure 1d–f).

Carbon‐nitrogen (C:N) ratios in *D*. *caespitosa* leaves exhibited pronounced seasonal changes (Figure 1g–i). C:N ratios were at their lowest during the time of new leaf emergence in late spring, but then rapidly increased until mid‐summer when they were at their highest. C:N ratios were not affected by treatment in neither the *induction* (treatment: *β* = 0.01, *SE* = 0.01, *t* = 0.78) nor the *response* phase (treatment: *β* = 0.02, *SE* = 0.01, *t* = 1.99).

### 4.3 Vole population densities and performance during the *response* phase

As is typical for vole populations in Kielder Forest during the trough of their cycle (Lambin et al., 1998), population densities in controls were much lower during the *response* phase (19–76 voles/ha) than the *induction* phase (37–206 vole/ha) (Figure 1a–c). Changes in population densities appeared to follow a similar pattern within pairs (Figure 1a–c).

**Figure 2:**
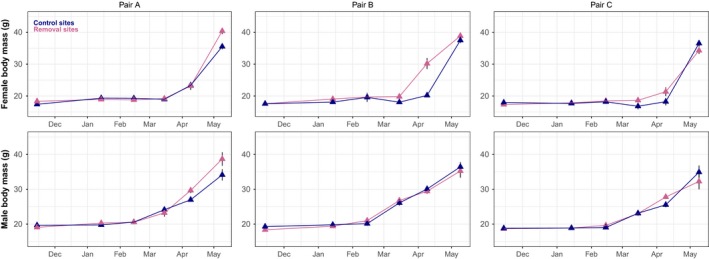
Changes in average female and male vole body mass (±*SE*) during the vole response phase of the experiment (November 2010–June 2011)

Body mass in both sexes increased over the spring months of the response phase (Figure 2) but was unaffected by the past density manipulations (treatment_males_: *β* = 0.04, *SE* = 0.81, *t* = 0.05; treatment_females_: *β* = −0.86, *SE* = 1.92, *t* = −0.45). No significant difference in the date of onset of spring reproduction was detected between the two treatment groups (*t*_treatment_ = −1.62). Spring population growth rates did not significantly differ between removals (0.17 ± 0.28 *SD*) and controls (0.09 ± 0.18 *SD*) (*t* = 1.36, *df* = 2, *p* = 0.31).

### 4.4 Vole survival

**Figure 3:**
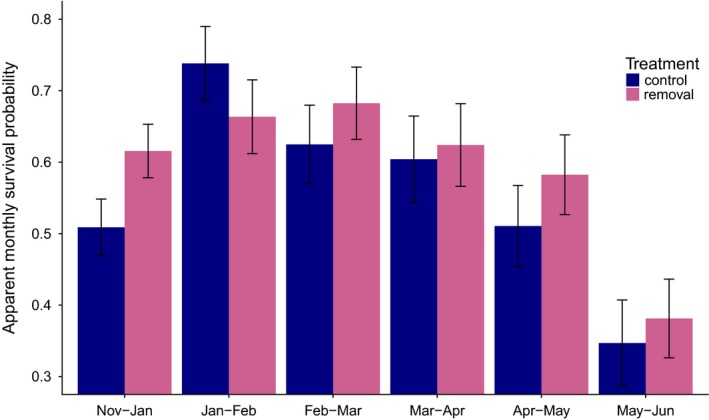
Monthly vole survival probabilities (±*SE*) on control and removal sites from November 2009 to June 2010

There was no evidence that apparent survival in spring of the response phase was affected by our manipulation treatment (Figure 3; see also Supporting Information Table S2). In the model that best supported the data (AICc weight = 0.80), survival *ɸ* depended on the interaction between “pair” and “date.” In the second best model (AICc weight = 0.17), *ɸ* depended on the interaction between “pair” and “date,” as well as “sex” and “date.” From the best model, survival probabilities varied from 0.33 (Pair B; May–June 2011) to 0.82 (Pair C; January–February 2011), while recapture probabilities depended on “pair” and “date,” and varied from 0.58 (Pair A; November 2010) to 0.96 (Pair A; January 2011).

## 5 DISCUSSION

Using a large‐scale manipulation of vole populations, we have demonstrated delayed density‐dependent induction of silicon defenses by field voles in a natural grassland ecosystem over the large spatial scales relevant to their population dynamics. We have performed this using an experimental approach through a replicated manipulation of natural populations. We also experimentally tested the hypothesis, in field conditions, that this silicon induction would affect the growth rates of female voles and delay the onset of their reproduction in the spring, thereby providing a nutritional explanation for the generation of multiyear population cycles of field voles in northern England (Reynolds et al., 2012). However, we found that the effects of the grazing on silicon induction were relatively short‐lived and clearly insufficient in both magnitude and duration to elicit effects on vole demography (Ergon et al., 2011). Furthermore, suppressing vole population densities did not have any effects on the timing of the onset of vole breeding in the following spring nor on the spring population growth rate, as expected given silicon levels were no longer elevated at that time. Previous empirical studies have documented such a lagged effect of density (Ergon et al., 2011), while modeling revealed that this is sufficient for causing cycles (Smith et al., 2006). Our findings do not support our hypothesis that this delayed density dependence, and hence potentially vole population cycles, is mediated by past grazing pressure increasing concentrations of silicon defense and reducing subsequent vole reproduction (Reynolds et al., 2012). However our study suggests that, in the field, vole densities may be insufficient to exceed the leaf damage thresholds required to induce silicon defenses to sufficient levels to impact on vole demography; the relatively low peak phase vole densities present during part of our study resulted in low leaf damage rates and relatively low levels of silicon induction (see below) in comparison with our previous greenhouse and field‐enclosure studies (Hartley & DeGabriel, 2016; Reynolds et al., 2012). On average, only 7.6% of leaves were found to be damaged in July 2010, the only time that damage was scored, well below the 20% foliar damage found to be required for silicon induction in the Reynolds et al. (2012) greenhouse experiment.

We predicted our vole density manipulations to create a divergence in mean leaf silicon concentrations between treatment groups and that the magnitude of this difference would depend on the age of the leaves and how long they had been damaged (Reynolds et al., 2012). Silicon concentrations were on average 22% higher on the control than on the removal sites over the summer and autumn of the *induction phase* in 2010 but only 4% higher over the preceding autumn and winter, confirming both our predictions. Furthermore, this time‐lagged silicon response to grazing treatment is consistent with the results of our greenhouse and field‐enclosure studies (Hartley & DeGabriel, 2016; Reynolds et al., 2012), as well those of Wieczorek, Zub, et al. (2015), although the validity of the conclusions drawn in the latter study has recently been questioned (Soininen, Hamel, & Yoccoz, 2017).

Although the magnitude of silicon induction in this experiment was relatively small compared with that observed in previous studies (Hartley & DeGabriel, 2016; Massey, Ennos, & Hartley, 2007a; Reynolds et al., 2012), it is similar to concentrations measured in *D*. *caespitosa* in another large‐scale field study in northern Norway (Soininen et al., 2013). In High Arctic Norway, vole populations fluctuate cyclically, but at much lower densities relative to Kielder Forest. In fact, vole densities on the control sites during the *induction phase* of this experiment were substantially lower than those typically seen, until recently, during cyclic peaks in Kielder Forest (200–765 voles/ha; Lambin et al., 2000; Ergon et al., 2011). An alternative view inspired by theoretical work is that the low amplitude cycles that prevailed in Kielder Forest at the time of our experiment are a transient embodiment of dampening cycles that could exist for some decades, even in the absence of the process that hitherto generated high amplitude cycles. If this conjecture was true, it would make experimental testing of the causes of vole population cycles very challenging (Lambin, Krebs, Moss, Stenseth, & Yoccoz, 1999). Our previous work has demonstrated that silicon induction in response to repeated damage on plants and over a threshold of 20% of plant biomass is sufficient to impact on vole performance (Massey & Hartley, 2006; Massey et al., 2007b; Reynolds et al., 2012). This work also demonstrated that if only 5% of foliage is removed, silicon induction is only of the order of around 20% and is short‐lived (2–3 months), exactly what we found in our field study, that is, a transient induction of 22%. This suggests that the level of grazing pressure achieved in our study lies to the left of the inflexion point of the silica‐grazing intensity relationship and, thus, is insufficient to cause substantial increases in silicon uptake in plants.

The novelty of our study was that we attempted to quantify the effects of natural vole grazing pressure on silicon induction in the field at the landscape scale; such grazing is likely to be more variable in both magnitude and frequency than when plants and voles are in a confined space in a laboratory‐based study, or in fenced enclosures. Furthermore, the induction of silicon defenses in natural grasslands may be influenced by spatial and temporal heterogeneity in factors other than the grazing history of individual plants. These factors can affect both silicon availability in the environment and silicon uptake by plants (Hartley & DeGabriel, 2016) and include phenotypic and genotypic plasticity within a species (Hartley, Fitt, McLarnon, & Wade, 2015; McLarnon, McQueen‐Mason, Lenk, & Hartley, 2017; Soininen et al., 2013), as well as abiotic factors such as temperature (Liang et al., 2006), soil type and pH (Quigley et al., 2017) , and precipitation (Quigley & Anderson, 2014).

Even though the induction of silicon in our field study may have been too small and transient to affect the timing of onset of spring reproduction, we expected voles to respond negatively to previously high population densities because other, nonplant based mechanisms exist by which high previous densities affect current populations, such as pathogen infection, intraspecific competition, and predation. However, contrary to our predictions, there were no detectable effects of reducing vole densities on subsequent vole mass and the timing of onset of spring breeding, possibly reflecting the declining populations of voles in Kielder Forest during our study as well as the dampening of cycles observed Europe‐wide (Cornulier et al., 2013), which led to both our control and removal sites having relatively low densities. The observed dynamics might therefore reflect the resonating impact of processes no longer operating at the time of the experiment. Even though average vole densities on the control sites were approximately 100 voles/ha higher than those on the removal sites during spring and early summer of the *induction phase*, the average spring densities we encountered (124 voles/ha) were substantially lower than the maximum spring estimates of 278 voles/ha observed in previous studies, and our densities were in a range of density with little obvious impact on the onset of vole reproduction in figure 2A of Ergon et al. (2011). This suggests that negative density‐dependent processes in vole populations only operate at higher densities than the ones reached by voles in the latest peak.

In conclusion, although we found landscape‐scale induction of silica defenses in grass in response to manipulating herbivore densities, this induction appears to be too small and transient to impact on vole demography. Hence, at the spatial scale and over the range of vole densities, this study was conducted, we did not find support for our hypothesis that silicon defenses in grasses drive the negative delayed density dependence of field vole populations in Kielder Forest. Long‐term studies would, however, be needed in order to test whether this conclusion is robust to varying vole population dynamics (i.e., cycles) and environmental conditions.

## CONFLICT OF INTEREST

The authors declare no conflict of interest.

## AUTHORS CONTRIBUTIONS

SH and XL designed the study, JD conducted the field work, and LR performed the analyses. All four authors wrote the manuscript.
