# Supplementary material for: Population‐level manipulations of field vole densities induce subsequent changes in plant quality but no impacts on vole demography
Source: Ecol Evol. 2018 Jul 13;8(16):7752–62. doi: 10.1002/ece3.4204 (PMC6145023; doi:10.1002/ece3.4204)
Supplement: Supplementary file 1 [file ECE3-8-7752-s001.docx]

**SUPPORTING INFORMATION**

**Ruffino et al. Population-level manipulations of field vole densities induce subsequent changes in plant quality but no impacts on vole demography. *Ecology and Evolution***

**Table S1.** Dates of plant sampling for silicon analyses, vole sign index (VSI) surveys, vole removal sessions and capture-mark-recapture surveys on both the control (C) and removal (R) sites of each pair. Dates refer to a time period of ± 3 days. Asterisks indicate that samples were also analysed for carbon-nitrogen ratios.

**Table S2**. Parameter estimates derived from the best survival model: phi = (pair)_date_, (.)_date_; p = (pair)_snow_, (.)_snow_

In this model, survival probabilities depended on the interaction between pair (3 pairs) and date (6 time intervals), while recapture probabilities depended on the interaction between pair and snow cover (presence vs. absence of snow during trapping session).

| **Parameter** | **Estimate** | **SE** |  | **95%LCI** | **95%UCI** |
| --- | --- | --- | --- | --- | --- |
| 1:Phi (Pair A; Nov-Jan) | 0.634 | 0.051 |  | 0.529 | 0.728 |
| 2:Phi (Pair A; Jan-Feb) | 0.647 | 0.066 |  | 0.510 | 0.764 |
| 3:Phi (Pair A; Feb-Mar) | 0.598 | 0.058 |  | 0.480 | 0.705 |
| 4:Phi (Pair A; Mar-Apr) | 0.601 | 0.065 |  | 0.470 | 0.719 |
| 5:Phi (Pair A; Apr-May) | 0.490 | 0.056 |  | 0.382 | 0.599 |
| 6:Phi (Pair A; May-Jun) | 0.412 | 0.069 |  | 0.286 | 0.550 |
| 7:Phi (Pair B; Nov-Jan) | 0.477 | 0.049 |  | 0.384 | 0.572 |
| 8:Phi (Pair B; Jan-Feb) | 0.620 | 0.070 |  | 0.478 | 0.745 |
| 9:Phi (Pair B; Feb-Mar) | 0.641 | 0.075 |  | 0.484 | 0.772 |
| 10:Phi (Pair B; Mar-Apr) | 0.679 | 0.075 |  | 0.519 | 0.805 |
| 11:Phi (Pair B; Apr-May) | 0.649 | 0.073 |  | 0.496 | 0.777 |
| 12:Phi (Pair B; May-Jun) | 0.335 | 0.062 |  | 0.227 | 0.465 |
| 13:Phi (Pair C; Nov-Jan) | 0.580 | 0.044 |  | 0.492 | 0.663 |
| 14:Phi (Pair C; Jan-Feb) | 0.815 | 0.054 |  | 0.685 | 0.899 |
| 15:Phi (Pair C; Feb-Mar) | 0.739 | 0.062 |  | 0.601 | 0.842 |
| 16:Phi (Pair C; Mar-Apr) | 0.565 | 0.078 |  | 0.410 | 0.708 |
| 17:Phi (Pair C; Apr-May) | 0.528 | 0.086 |  | 0.362 | 0.688 |
| 18:Phi (Pair C; May-Jun) | 0.353 | 0.087 |  | 0.206 | 0.534 |
| 19:Phi (Nov-Jan) | 0.564 | 0.028 |  | 0.510 | 0.617 |
| 20:Phi (Jan-Feb) | 0.699 | 0.037 |  | 0.621 | 0.766 |
| 21:Phi (Feb-Mar) | 0.655 | 0.037 |  | 0.579 | 0.724 |
| 22:Phi (Mar-Apr) | 0.614 | 0.042 |  | 0.530 | 0.693 |
| 23:Phi (Apr-May) | 0.547 | 0.040 |  | 0.468 | 0.624 |
| 24:Phi (May-Jun) | 0.366 | 0.041 |  | 0.290 | 0.449 |
| 25:p (Pair A; Snow present) | 0.578 | 0.107 |  | 0.366 | 0.764 |
| 26:p (Pair A; Snow absent) | 0.961 | 0.022 |  | 0.887 | 0.987 |
| 27:p (Pair B; Snow present) | 0.901 | 0.092 |  | 0.544 | 0.986 |
| 28:p (Pair B; Snow absent) | 0.920 | 0.034 |  | 0.823 | 0.966 |
| 29:p (Pair C; Snow present) | 0.903 | 0.065 |  | 0.687 | 0.975 |
| 30:p (Pair C; Snow absent) | 0.918 | 0.032 |  | 0.831 | 0.963 |
| 31:p (Snow present) | 0.776 | 0.058 |  | 0.642 | 0.869 |
| 32:p (Snow absent) | 0.934 | 0.017 |  | 0.892 | 0.960 |
